# Ultrasensitive Detection of GRP78 in Exosomes and Observation of Migration and Proliferation of Cancer Cells by Application of GRP78-Containing Exosomes

**DOI:** 10.3390/cancers14163887

**Published:** 2022-08-11

**Authors:** Naoko Tsurusawa, Kanako Iha, Akane Sato, Hsin-Yi Tsai, Hikaru Sonoda, Satoshi Watabe, Teruki Yoshimura, Deng-Chyang Wu, Ming-Wei Lin, Etsuro Ito

**Affiliations:** 1 Department of Biology, Waseda University, Shinjuku, Tokyo 162-8480, Japan; 2 Department of Medical Research, E-Da Hospital/E-Da Cancer Hospital, Kaohsiung 82445, Taiwan; 3 School of Pharmacy, Kaohsiung Medical University, Kaohsiung 80708, Taiwan; 4 Hakarel Inc., Ibaraki, Osaka 567-0085, Japan; 5 Waseda Research Institute for Science and Engineering, Waseda University, Shinjuku, Tokyo 169-8555, Japan; 6 School of Pharmaceutical Sciences, Health Sciences University of Hokkaido, Tobetsu, Hokkaido 061-0293, Japan; 7 Division of Gastroenterology, Department of Internal Medicine, Kaohsiung Medical University Hospital, Kaohsiung 80756, Taiwan; 8 Regenerative Medicine and Cell Therapy Research Center, Kaohsiung Medical University, Kaohsiung 80756, Taiwan; 9 Department of Nursing, College of Medicine, I-Shou University, Kaohsiung 82445, Taiwan; 10 Graduate Institute of Medicine, Kaohsiung Medical University, Kaohsiung 80708, Taiwan

**Keywords:** cancer stemness, exosome, cultured gastric cancer cell, GRP78, ultrasensitive ELISA

## Abstract

**Simple Summary:**

Cancer cells release exosomes to their surrounding cells, and it is believed that trace amounts of proteins included in exosomes promote cancer stemness. In the present study, we note 78-kDa glucose-regulated protein (GRP78), which is involved in cancer progression, and present the protocol for measurements of trace amounts of GRP78 in exosomes released from cultured gastric cancer cells using an ultrasensitive ELISA with thio-NAD cycling. We found that when high-GRP78-containing exosomes were incubated with cultured cancer cells, these cells increased their stemness, for example, an increase in indices of both an MTT assay and a wound healing assay. The technique for quantifying proteins in exosomes described here will advance our understanding of cancer stemness progression via exosomes.

**Abstract:**

Cancer cells communicate with each other via exosomes in the tumor microenvironment. However, measuring trace amounts of proteins in exosomes is difficult, and thus the cancer stemness-promoting mechanisms of exosomal proteins have not been elucidated. In the present study, we attempted to quantify trace amounts of 78-kDa glucose-regulated protein (GRP78), which is involved in cancer progression, in exosomes released from cultured gastric cancer cells using an ultrasensitive ELISA combined with thio-NAD cycling. We also evaluated the cancer stemness-promoting effects by the application of high-GRP78-containing exosomes to cultured gastric cancer cells. The ultrasensitive ELISA enabled the detection of GRP78 at a limit of detection of 0.16 pg/mL. The stemness of cancer cultured cells incubated with high-GRP78-containing exosomes obtained from GRP78-overexpressed cells was increased on the basis of both an MTT assay and a wound healing assay. Our results demonstrated that the ultrasensitive ELISA has strong potential to measure trace amounts of proteins in exosomes. Further, exosomes with a high concentration of GRP78 promote the cancer stemness of surrounding cells. The technique for quantifying proteins in exosomes described here will advance our understanding of cancer stemness progression via exosomes.

## 1. Introduction

Cancer stem cells have recently attracted particular attention in cancer treatment [1]. If even a small number of cancer stem cells remain in the body after surgery or chemotherapy, they are capable of forming tumors similar to the original one [2] by self-renewal and asymmetric cell division [1]. Cancer stem cell formation is thought to be related to the tumor microenvironment, such as hypoxia and undernutrition, resulting in the onset of cancer invasion and metastasis [3,4]. Recent studies revealed that cancer cells also dedifferentiate into cancer stem cells [5,6]. That is, cancer cells can change their characteristics according to changes in the surrounding environment and their communication with other cells to promote cancer stemness and progression. Therefore, elucidation of the behavior and properties of cancer stem cells is an important issue in cancer research.

One way cancer cells communicate is through the exchange of exosomes [7]. The diameter of an exosome is approximately 100 nm [8]. Exosomes contain nucleic acids and proteins inside a lipid membrane, while membrane proteins are located on the membrane surface [9,10]. These molecules can be transported in a close or far range to achieve cell–cell communication [11]. In the tumor microenvironment, many cytokines and inflammatory substances such as chemokines are exchanged between cancer cells. Exosomes are also exchanged, which is thought to promote cancer malignancy [12]. Although proteins, not nucleic acids, should be evaluated in exosomes, it is often difficult to detect trace amounts unless an enormous volume of exosomes can be collected [13]. A recently developed ultrasensitive enzyme-linked immunosorbent assay (ELISA), which was improved by Iha et al., can detect proteins at the zeptomole level [14,15], and thus this ultrasensitive ELISA is expected to be applicable for the detection of proteins in exosomes.

We must consider which protein in exosomes should be studied. In cancer, rapid cell proliferation creates an environment that induces endoplasmic reticulum (ER) stress, such as hypoxia and nutrient starvation [16]. Some proteins that are normally involved in the ER stress response are overexpressed in cancer cells [16]. One of these proteins is a 78-kDa glucose-regulated protein (GRP78), which is a member of the heat shock protein 70 (HSP70) family that exists only in the ER in normal cells where it acts as an ER molecule chaperone and an ER stress sensor [17]. GRP78 is overexpressed in cancer cells, and its expression is observed in cell regions such as cell membrane, cytoplasm, mitochondria, nucleus, and cell secretions, as well as in the ER [18]. It is also involved in tumor cell proliferation, resistance to apoptosis, avoidance of the immune response, invasion, metastasis, and angiogenesis [17]. GRP78 functions under stress, such as hypoxia, glucose deficiency, and tumor microenvironment [17]. Although GRP78 is overexpressed in solid gastric tumors, there is no reliable biomarker for early prognosis in the serum of gastric cancer patients.

In the present study, we measured GRP78 concentrations in the exosomes of cultured gastric cancer cells using the ultrasensitive ELISA combined with thionicotinamide-adenine dinucleotide (thio-NAD) cycling. We hypothesized that the application of high-GRP78-containing exosomes to cultured gastric cancer cells would increase their stemness as examined by a 3-(4,5-di-methylthiazol-2-yl)-2,5-diphenyltetrazolium bromide, yellow tetrazole (MTT) assay and a wound healing assay.

## 2. Materials and Methods

### 2.1. Cell Culture

Human gastric cancer cell lines AGS (CRL-1739, ATCC) and MKN45 (ACC-409, DSMZ) were purchased from ATCC and DSMZ, respectively, and MKN45 was also obtained from JCRB Cell Bank (JCRB0254 MKN45). Four kinds of GRP78-transfected cells were purchased from the National RNAi Core Facility (RNA Technology Platform and Gene Manipulation Core, Academia Sinica, Taipei, Taiwan) as follows: (1) AGS/nc (AGS with scrambled shRNA, shLacZ1339, shLacZ, clone ID: TRCN231722; referred to as ‘GRP78-mock AGS’ hereafter). (2) AGS/GRP78+ (AGS with GRP78-Bip-pLAS2w; referred to as ‘GRP78-overexpressed (OE)’ AGS hereafter). (3) MKN45/nc (MKN45 with scramble shRNA, shLacZ1339, shLacZ, clone ID: TRCN231722; referred to as ‘GRP78-mock MKN45’ hereafter). (4) MKN45/GRP78+ (MKN45 with GRP78-Bip-pLAS2w; referred to as ‘GRP78-OE MKN45’ hereafter). All the cells were cultured using RPMI1640 (Thermo Fisher Scientific, Gibco, Waltham, MA, USA or Nacalai Tesque, Kyoto, Japan) supplemented with 10% fetal bovine serum and penicillin–streptomycin mixed solution (Gibco or Nacalai Tesque) under 5% CO_2_ at 37 °C; for the transfected cells, 0.75 mg/mL puromycin dihydrochloride (Cat # ant-pr-1, InvivoGen, San Diego, CA, USA) was added.

### 2.2. Exosome Isolation

Exosomes were extracted from gastric cancer cell culture media using Total Exosome Isolation Reagent (from cell culture media; Cat # 4478359, Thermo Fisher Scientific, Invitrogen, Waltham, MA, USA). The culture media were centrifuged at 2000× *g* for 30 min at 4 °C to remove cells and cell debris. The supernatant was filtered through a 0.22 µm filter and ultra-filtered to remove material below 100 kDa. A half-volume of an exosome isolation reagent was added to the concentrated sample before incubating overnight at 4 °C. The samples were centrifuged at 10,000× *g* for 60 min at 4 °C. The exosome-containing pellet was resuspended in PBS.

### 2.3. Ultrasensitive Thio-NAD Cycling ELISA

**Figure 1:**
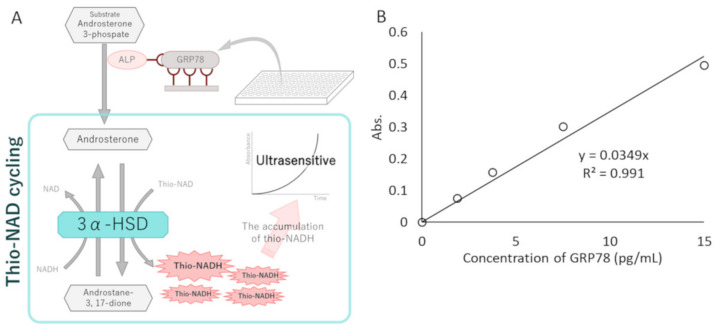
GRP78 measurements using an ultrasensitive ELISA with thio-NAD cycling. (**A**) Schematic representation of an ultrasensitive ELISA with thio-NAD cycling. A conventional sandwich ELISA was combined with a thio-NAD cycling assay, producing signals in a quadratic-function-like response (i.e., triangle number) over time. This system comprised ALP linked with a secondary antibody against the target protein, an androsterone derivative as the first substrate, 3α-HSD as the enzyme for thio-NAD cycling, and their coenzymes (NADH and thio-NAD). Thio-NADH accumulation was measured as absorbance at 405 nm. (**B**) A typical linear calibration curve of GRP78 standard proteins (i.e., antigen) from the ultrasensitive thio-NAD cycling ELISA. The absorbance was obtained from a 60-min cycling reaction time. The antigen was applied in the range of 1.89–15.0 pg/mL.

Measurements of GRP78 in exosomes were performed with the ultrasensitive thio-NAD cycling ELISA [19,20]. This ELISA is coupled with a thio-NAD cycling reaction, and thus the signal obtained from a sandwich ELISA using a primary and secondary antibody is amplified by the thio-NAD cycling reaction [21,22] (Figure 1A). For ultrasensitive detection of GRP78, we used a GRP78 ELISA kit (Human HSPA5/GRP78/BiP (sandwich ELISA) ELISA Kit; LS-F11578, LifeSpan Biosciences, Seattle, WA, USA) and thio-NAD cycling. Briefly, the GRP78 standards contained in the kit and the exosome samples were incubated in primary GRP78 antibody-bound 96-well plates for 2 h at 37 °C. The total protein concentrations in the exosomes collected from AGS/MKN45 GRP78-mock and OE were adjusted to 15 μg/100 μL using a Bicinchoninic Acid Protein Assay (BCA) kit (Cat # 23227, Thermo Fisher Scientific, Waltham, MA, USA). The wells were incubated with the secondary GRP78 antibody for 1 h at 37 °C. Following this procedure, alkaline phosphatase (ALP) was linked with the secondary antibody via a biotin–streptavidin reaction for 1 h at room temperature. To amplify the ELISA signal, 100 μL of thio-NAD cycling solution (containing 1.0 mM NADH, 2.0 mM thio-NAD, 0.4 mM 17β-methoxy-5β-androstan-3α-ol 3-phosphate (provided by one of the authors, T.Y.), and 10 U/mL 3α-hydroxysteroid dehydrogenase (3α-HSD; T-58, Asahi Kasei Pharma, Tokyo, Japan) in 100 mM Tris-HCl, pH 9.0 [23,24]) was added to each well. Thio-NADH was measured with a microplate reader (Corona Electric SH-1000) at 405 nm. The 405 nm signals were normalized to those at 660 nm [25,26]. For the linear calibration curves, the experimental data were obtained by subtracting the mean value of the blank signals from each of the corresponding measured datapoints (see Figure 1B).

### 2.4. Nanoparticle Tracking Analysis

Exosome pellets were suspended by a 100 µL solution of PBS, and then diluted 1:1000 with PBS. The exosome size was examined with a nanoparticle tracking video-microscope, ZetaView (Particle Metrix, Inning am Ammersee, Germany) using ZetaView software v8.05.12 SP1 (Particle Metrix, Inning am Ammersee, Germany).

### 2.5. Western Blotting

Protein concentrations of exosome samples were measured using a BCA kit. The total protein content (10 µg) was separated by Extra PAGE One Precast Gel 5%–15% (Cat # 13061-94, Nacalai Tesque, Kyoto, Japan) and transferred onto polyvinylidene fluoride membranes (PVDF; EMD Millipore, Burlington, MA, USA). The membrane was blocked with 3% BSA in TBS with Tween 20 (TBS-T) for 1 h at room temperature and then incubated overnight at 4 °C with a 1:2500 solution of primary antibody (anti-CD63 antibody; Cat # ab134045, Abcam, Cambridge, UK). After washing with TBS-T, horseradish-peroxidase-conjugated anti-rabbit antibody (1:2000; Cat # 7074, Cell Signaling Technology, Danvers, MA, USA) was applied as the secondary antibody for immunostaining and incubated for 1 h at room temperature. After washing with TBS-T, a chemiluminescent substrate (WP20005, ECL Chemiluminescent Substrate Reagent Kit, Invitrogen, Novex, Waltham, MA, USA) was applied to detect bands. Densitometry was performed using LAS-3000 (FujiFilm, Tokyo, Japan).

Cells were collected and washed with PBS. The total protein samples were extracted, and protein concentrations were measured using Bio-Rad Bradford Protein Assays (Bio-Rad, Hercules, CA, USA). Equal quantities of total protein were separated by electrophoresis on BOLT BISTRIS PLUS 4%–12% SDS-PAGE (Thermo Scientific, Waltham, MA, USA) and transferred onto PVDF membranes. The membranes were incubated in blocking buffer (Bio-Rad, Hercules, CA, USA) for 30 min at room temperature and then overnight at 4 °C with the primary antibody of the anti-GRP78 antibody (1:1000; Cat # 3177S, Cell Signaling Technology, Danvers, MA, USA) or anti-actin antibody (1:1000; Cat # MAB1501, Merck Millipore, Burlington, MA, USA). After washing with PBS containing Tween 20 (PBS-T), the horseradish-peroxidase-conjugated anti-rabbit antibody (1:5000; Cat # NA934, GE Healthcare, Chicago, IL, USA) or horseradish-peroxidase-conjugated anti-mouse antibody (1:20,000; Cat # NA931, GE Healthcare, Chicago, IL, USA) was applied as the secondary antibody for immunostaining and incubated for 2 h at room temperature. After washing with PBS-T, a chemiluminescent substrate (Cat # WBKLS0500, Merck Millipore, Burlington, MA, USA) was applied to detect bands. The images were analyzed with ImageJ (NIH).

### 2.6. MTT Assay

AGS and MKN45 cells were seeded at 1 × 10^4^ cells per well in 96-well culture plates with 100 µL medium and incubated overnight at 37 °C. Exosome samples without phenol red medium were added to the cells. After 48 h, 10 µL of an MTT labeling reagent in an MTT Cell Proliferation and Cytotoxicity Assay Kit (Cat # AR1156, Boster, Pleasanton, CA, USA) was added into 96-well plates, and the cells were incubated for 4 h at 37 °C. The 100 µL solubilization buffer in the kit was then added to each of the wells, and the plates were incubated overnight at 37 °C. The absorbance was measured at a 570 nm wavelength with a microplate reader.

### 2.7. Flow Cytometry

AGS and MKN45 cells were prepared as a single cell suspension for staining. For surface staining, the following antibodies were purchased from BD Pharmingen (Franklin Lakes, NJ, USA): PE-conjugated mouse anti-human CD44, PE-conjugated mouse IgG2b, FITC mouse anti-human CD24, and FITC mouse IgG2a. AGS and MKN45 cells were harvested to 6-well plates and incubated overnight. The cells were treated for 3 days with exosomes containing serum-free medium. After treatment, the cells were collected and incubated for 30 min on ice with a 1:10 solution of the antibodies in a staining buffer. After staining, the cells were filtered through a 38 µm cell strainer. The data were acquired by a flow cytometer (BD Accuri C6 Plus; BD Biosciences, Franklin Lakes, NJ, USA).

### 2.8. Wound Healing Assay

AGS or MKN45 of 3.0 × 10^4^ cells were plated in a 2-well cell culture insert (ibidi, Gräfelfing, Germany) on a plate and incubated at 37 °C overnight for AGS and for 2 days for MKN45. After incubation, the culture cell inserts were removed from the plate, and the medium containing exosome samples was treated. The data are representative of 3 independent experiments.

### 2.9. Statistical Analysis

All data are represented as the mean ± standard deviation of at least 3 independent experiments. The statistical significance of differences was assessed with the Welch *t*-test, or by 1-way ANOVA and the post hoc Holm test (*R*-4.2.1). *p* values < 0.05 were considered statistically significant. The limit of detection (LOD) of the ultrasensitive thio-NAD cycling ELISA was estimated from the mean of the blanks, the standard deviation (SD) of the blanks, and a confidence factor of 3. The limit of quantitation (LOQ) was estimated by the same method used to estimate the LOD, but with a confidence factor of 10. The coefficients of variation (CVs) for GRP78 standards were obtained in the assessments of inter-assay reproducibility.

## 3. Results

### 3.1. Ultrasensitive Determination of GRP78

The ultrasensitive thio-NAD cycling ELISA was applied to obtain the LOD and LOQ for the GRP78 standard protein (Figure 1B). The GRP78 standards (ranging from 1.88 to 15.0 pg/mL) and the sample buffer contained in the GRP78 ELISA kit (see Section 2) were used to obtain the linear calibration curves for GRP78. The absorbance of thio-NADH was measured after 60 min of thio-NAD cycling. A typical linear calibration curve was expressed as *y* = 0.0349*x*, *R*^2^ = 0.991 (Figure 1B). The LOD calculated from this calibration curve was 1.99 × 10^−19^ moles/assay (0.16 pg/mL), and the minimum LOQ was 6.62 × 10^−19^ moles/assay (0.52 pg/mL). The molecular mass of GRP78 was 78.3 kDa, and the assay volume was 100 μL. The intra-assay CV was 1.1% for 10 pg/mL (*n* = 3). These values indicate approximately 9000 times higher sensitivity than the ELISA method typically used for measuring GRP78 [27].

### 3.2. Characterization of Isolated Exosomes by Western Blotting and Nanoparticle Tracking Analysis

**Figure 2:**
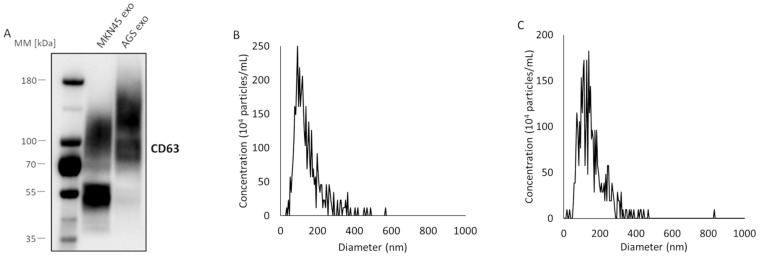
Characterization of exosomes isolated from the supernatant of cultured gastric cancer cells using a combination of a polymer precipitation method and ultra-filtration method. (**A**) Western blotting of an exosome-marker protein, CD63, for exosomes in the supernatant of MKN45 and AGS cells. The molecular mass of CD63 is 40–50 kDa, and CD63 forms a dimer and is glycosylated. (**B**) Nanoparticle tracking analysis for exosomes in the supernatant of AGS cells. The peak value was around 93 nm. (**C**) Nanoparticle tracking analysis for exosomes in the supernatant of MKN45 cells. The peak value was around 138 nm.

The exosome samples were collected from the supernatant of cultured gastric cancer cells (AGS and MKN45) using a polymer precipitation method and an ultra-filtration method. Western blotting was performed for the exosome-marker protein CD63 (Figure 2A). The molecular mass of CD63 is 40–50 kDa, but it forms a dimer and is glycosylated [28,29]. Thus, a broad band appeared at a position higher than the original band (Figure 2A), confirming that the vesicles obtained from both AGS and MKN45 were exosomes. Next, nanoparticle tracking analyses were performed for the exosomes collected from AGS (Figure 2B) and MKN45 (Figure 2C). The diameters of most of the vesicles were approximately 100 nm in both AGS and MKN45, supporting that the vesicles were exosomes [8].

### 3.3. Effects of High-GRP78-Containing Exosomes on Profiles of Untreated Cultured Gastric Cancer Cells

**Figure 3:**
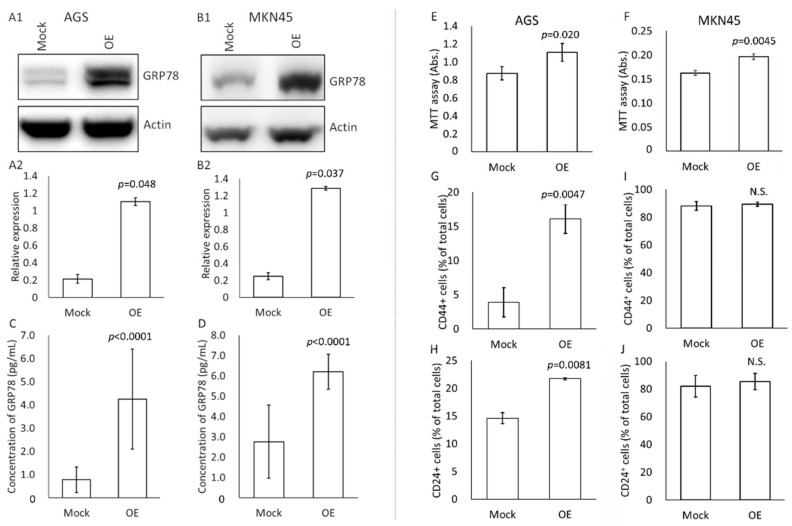
Effects of the application of GRP78-containing exosomes on changes in profiles of cultured gastric cancer cells. (**A**,**B**) Western blotting for GRP78 using cell disruption solutions of AGS (**A1**) and MKN45 (**B1**) mock and GRP78-overexpressed (OE) cells. Relative expression levels of GRP78 compared with actin expression in AGS (**A2**) and MKN45 (**B2**) mock and GRP78-OE cells. *n* = 3 each. (**C**) Thio-NAD cycling ELISA for GRP78 in exosomes derived from AGS mock and GRP78-OE cells. *n* = 3 each. (**D**) Thio-NAD cycling ELISA for GRP78 in exosomes derived from MKN45 mock and GRP78-OE cells. *n* = 4 each. (**E**) MTT assays for cell viability of AGS cells by application of exosomes isolated from AGS mock and GRP78-OE cells. *n* = 4 each. (**F**) MTT assays for cell viability of MKN45 cells by application of exosomes isolated from MKN45 mock and GRP78-OE cells. *n* = 3 each. (**G**,**H**) Flow cytometry assays (stemness measurements) for (**G**) CD44-positive and (**H**) CD24-positive cells in AGS by application of exosomes isolated from AGS mock and GRP78-OE cells. *n* = 3 each. (**I**,**J**) Flow cytometry assays (stemness measurements) for (**I**) CD44-positive and (**J**) CD24-positive cells in MKN45 cells by application of exosomes isolated from MKN45 mock and GRP78-OE cells. *n* = 3 each.

We examined how high-GRP78-containing exosomes changed the profile of cultured gastric cancer cells (AGS and MKN45) (Figure 3). First, the amount of GRP78 was observed by Western blotting for cell crushing solutions of GRP78-mock and GRP78-OE AGS and MKN45 cells (Figure 3A,B). In both cases of AGS and MKN45, GRP78 was sufficiently overexpressed in the OE cells compared with the mock cells.

To further confirm the GRP78 expression level in the exosomes derived from AGS/MKN45 GRP-78 mock and OE cells, we applied our ultrasensitive thio-NAD cycling ELISA (Figure 3C,D). The exosomes contained the following amounts of GRP78: 5.2 fg in 1 µg exosomes derived from AGS GRP78-mock cells, 21.0 fg in 1 µg exosomes derived from AGS GRP78-OE cells, 280 fg in 1 µg exosomes derived from MKN45 GRP78-mock cells, and 620 fg in 1 µg exosomes derived from MKN45 GRP78-OE cells. That is, the amount of GRP78 in exosomes considerably differed between cell types.

**Figure 4:**
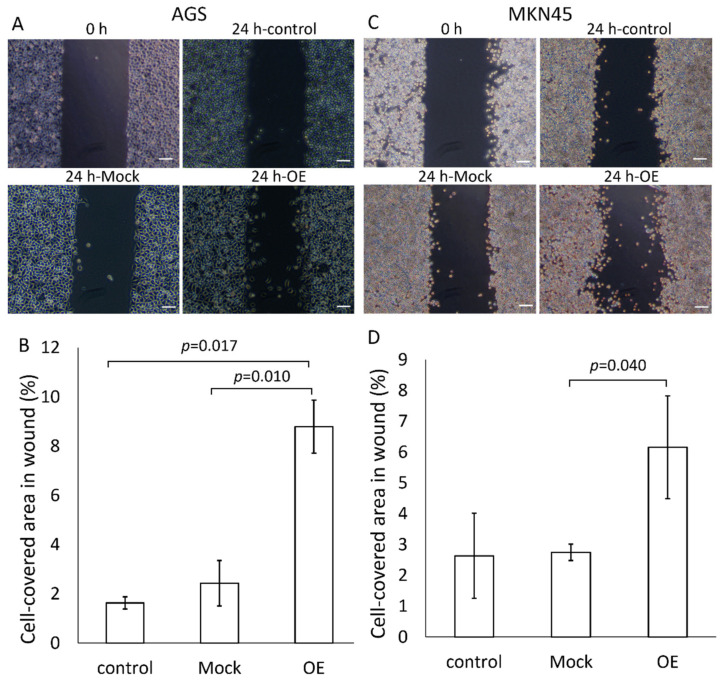
Wound healing assays (migration capability and proliferation capability) of AGS and MKN45 after treatment with GRP78-containing exosomes. (**A**) Typical pictures 24 h later of wound healing assays for AGS after application of exosomes derived from AGS mock and GRP78-OE cells. For control experiments, PBS was used instead of GRP78-containing exosomes. Scale bars, 100 μm. (**B**) Typical pictures 24 h later of wound healing assays for MKN45 after application of exosomes derived from MKN45 mock and GRP78-OE cells. For control experiments, PBS was used instead of GRP78-containing exosomes. Scale bars, 100 μm. (**C**,**D**) Summarized data for (**A**) and (**B**), respectively. *n* = 3 each.

Second, an MTT assay was performed to examine the cell viability and proliferation of AGS and MKN45 cells by applying GRP78-containing exosomes originating from mock and OE cells (Figure 3E,F). The total protein concentration used was 2 μg/mL (one well was 100 μL), and the application period was 2 days. Exosomes released from cancer cells may change the cell viability and proliferation rate, and thus overdosing the application concentrations to the cells might cause overly drastic changes [30]. If the total protein amount of the exosomes was higher than 2 μg/mL, the cells proliferated too quickly, and no difference was observed. If the amount was less than 2 μg/mL, no significant differences were detected between the two conditions (i.e., mock and OE). We thus considered that the total protein amount of 2 μg/mL was suitable to examine the function of GRP78 in exosomes in this assay as well as in the following wound healing assay (see Figure 4). In addition, we considered the application period of 2 days reasonable as the amount of culture medium was small and the cells were cultivated in serum-free medium, and the cells were starved for nutrients if the period was extended beyond 2 days. If the period was shorter than 2 days, no significant differences were detected between the two conditions because the cell proliferation period was too short. Four experiments were performed for AGS and 3 for MKN45. For the both cell types, the absorbance of cells applied with the exosomes obtained from GRP78-OE cells was significantly higher than that of cells applied with the mock-derived exosomes (Figure 3E,F), indicating that the exosomes from GRP78-OE AGS/MKN45 cells increased the cell viability and proliferation capability.

Third, the stemness of AGS and MKN45 cells was examined by a flow cytometry assay for CD44 and CD24 after applying exosomes containing GRP78 (Figure 3G–J). The exosome concentrations were 50 μg/mL for AGS and 30 μg/mL for MKN45 (one well was 2 mL), and the application period was 2 days. If the exosome concentrations were smaller, no differences were detected between the two conditions. The expression of cancer stem cell markers appears to be a specific property of cells with high cancer stem cell capacity, and thus their changes were observed only when more exosomes were applied compared with the MTT assay (see Figure 3E,F). The application period of 2 days was considered reasonable for the same reasons as for the MTT assay. Three experiments were performed for each cell type. Significantly more CD24-positive AGS cells were observed when GRP78-OE AGS-derived exosomes were added than when the GRP78-mock cell-derived exosomes were added (Figure 3H). On the other hand, we detected no notable results using MKN45 (Figure 3I,J).

### 3.4. Change in Migration and Proliferation of Cultured Gastric Cancer Cells by the Application of GRP78-Containing Exosomes

A wound healing assay was performed to examine the cell migration and proliferation as cancer stem cell abilities in AGS and MKN45 cells (Figure 4). Exosomes derived from GRP78-mock AGS and GRP78-OE AGSs were treated with AGS cells at 2 µg/mL of the total quantity of proteins for 1 day (one well was 2 mL) (Figure 4A). The quantity of total proteins of exosomes applied to the cells was adjusted according to the results of the MTT assay (Figure 3E). The reason for the 1-day application period was as follows: the small silicon insert was small enough to become confluent with cells if the period was longer, and a period of less than 1 day was not long enough for cell migration and proliferation. Three experiments were performed. The addition of GRP78-OE AGS exosomes significantly increased cell proliferation, migration, and infiltration compared with GRP78 low-content exosomes (Figure 4B). The same results were obtained for MKN45 (Figure 4C,D). The results clearly demonstrated that the addition of GRP78-rich exosomes to untreated AGS/MKN45 cells increased cancer stem cell abilities such as cell migration and proliferation.

## 4. Discussion

The present results, obtained by successfully measuring trace amounts of GRP78 in exosomes using the ultrasensitive thio-NAD cycling ELISA, revealed for the first time that GRP78 contained in exosomes promotes the stemness of cancer cells. Cancer progresses in a tumor microenvironment in which cancer cells exposed to nutrient starvation, hypoxia, and chemotherapy may communicate with each other [31,32,33]. Recent studies suggest the existence of a wide variety of cells in cancer lesions that communicate with each other [34,35]. Once some signals are released extracellularly, however, it is difficult to track which cells release the signals and which cells receive the signals because the concentration of the signals is extremely low. Therefore, our proposed ultrasensitive ELISA combined with thio-NAD cycling can be widely applied for exosome studies.

Our target protein, GRP78, localizes in the ER and acts as a molecular chaperone in normal cells, whereas, in cancer, it is overexpressed in relation to the ER stress response and its localization changes. Especially in gastric cancer, GRP78 is highly expressed, and is thus reported to be a biomarker of gastric cancer [18]. The exosome-mediated release of GRP78 was reported by Li et al. [36]. Exosomes containing GRP78 are released in colon cancer, and one regulatory mechanism of GRP78 in exosomes originates from its deacetylation [36]. The release of GRP78 via exosomes, however, has not yet been confirmed in gastric cancer. In the present study, we confirmed that gastric cancer releases exosomes containing GRP78 (see Supplementary Materials). The mechanism underlying the fluctuating amounts of GRP78 in gastric cancer-derived exosomes has not been clarified, but as reported by Li et al. [36], deacetylation may be involved.

We strongly believe that it is so important that in vitro experiments should be analyzed carefully, like in the present study. Then, to provide a bridge to in vivo experiments, in the Supplementary Materials, we would like to show that the concentration of GRP78 in exosomes collected from patients’ specimens can be measured as was carried out in cultured cells. We can speculate how GRP78 affects cancer stemness as follows: GRP78 can position on plasma membrane as a multifunctional cell surface receptor for activated α2-macroglobulin [37]. Then, the interaction between GRP78 and α2-macroglobulin promotes the activation of ERK1/2, JNK, PI3K, Akt, NF-κB, and UPR in prostate cancer cells [37,38,39]. The activation of ERK1/2 and JNK enhances cell survival and proliferation, and the activation of Akt, NF-κB, and UPR promotes anti-apoptotic signals resulting in the induction of cell survival and proliferation. However, this is only speculation. We need to examine this topic in the future.

We shall compare the GRP78 concentration in the present in vitro experiments using the cancer cell lines and that in the human cancer serum specimens shown in the Supplementary Materials. The concentration of GRP78 in in vitro experiments was about 4 pg/mL as shown in Figure 3C, whereas that in in vivo specimens was about 10 pg/mL as shown in the Supplementary Materials. Interestingly, when we calculate the amount of GRP78 in the in vitro experiments with the amount mentioned in Figure 3C,D, it is 1–20 pg/mL, similar to the data of the in vivo specimens. That is, our concentration of GRP78 in the in vitro studies was reasonable.

Although exosome-mediated stemness promotion of cancer cells was recently reported, it was mostly thought to occur due to nucleic acids in exosomes [12,40]. We clarified that the promotion of cancer stemness occurs due to proteins (GRP78) in exosomes. Exosomes contain many proteins, for example heat shock protein family members and proteins inhibiting the immune system [41,42]. Therefore, our ultrasensitive ELISA technique is useful for studies of cell–cell communication by proteins in exosomes. A similar phenomenon of GRP78 in exosomes has been reported [43]. RNA interference of GRP78 in exosomes sensitized Sorafenib-resistant cancer cells to Sorafenib and reversed the drug resistance.

Why do we have to develop an ultrasensitive detection method? There are two important reasons, as follows: (1) We will be able to measure that which we have not been able to measure before; (2) We will reduce time and cost, and further we are able to reduce the quantity of specimens to be collected, which in turn reduces the burden on the patients. The present study provides the second benefit. As we have already mentioned, the measuring sensitivity in our ultrasensitive ELISA is approximately 9000 times higher than a conventional ELISA method for GRP78. For example, in our ultrasensitive ELISA we can measure the proteins at 5 μg/assay. This costs only about USD 2 for medium and wells, whereas a conventional method needs 9000 × 5 μg/assay, leading to a cost of about USD 18,000. This difference in cost is substantial. Furthermore, the time spent to retrieve exosomes is also significantly different. Thus, we need an ultrasensitive detection method.

The present results showed that the promotion of cancer stemness by the application of high-GRP78-containing exosomes is dependent on the cell type. MKN45 cells were reported to be more stem-cell-oriented (i.e., CD44 positive) in nature than other gastric cancer cells [44]. Ibuprofen, one of nonsteroidal anti-inflammatory drugs, suppresses the promotion of stemness in cancer. This drug suppressed the promotion of stemness in AGS, but this was barely observed in MKN45. That is, MKN45 is stem-cell-oriented [45]. Sphere forming is a property of stemness. MKN45 cells more easily formed spheres than AGS cells [46]. This also shows that MKN45 is stem-cell-oriented. Furthermore, the expression of Oct4 (a cancer stem cell marker) and the infiltration ability of MKN45 are higher than in AGS [47]. These findings suggest that MKN45 cells have high stemness [48,49], and thus the findings of the high ratio of CD44+ and CD24+ cells seem reasonable.

## 5. Conclusions

In the present study, we noted GRP78, which is involved in cancer progression, and presented the protocol for measurements of trace amounts of GRP78 in exosomes released from cultured gastric cancer cells using an ultrasensitive ELISA with thio-NAD cycling. We found that when high-GRP78-containing exosomes were incubated with cultured cancer cells, these cells increased their stemness, for example, an increase in indices of both an MTT assay and a wound healing assay.

In the future, proteins in exosomes may be used as cancer diagnostic markers. The quantification of proteins in exosomes contained in a small quantity of patient serum samples will provide a relatively noninvasive method of diagnosing cancer and its prognosis. In addition, quantification of exosomal proteins, such as GRP78, may be indispensable for elucidating the mechanism of cancer progression, because the scenario of this progression is not yet clarified [50,51,52]. We strongly believe that the present technique will provide detailed insight into the tumor microenvironment and cell–cell communication and contribute to elucidating the mechanisms of cancer progression.

## Data Availability

Data are contained within the article.

## Supplementary Materials

The following supporting information can be downloaded at: https://www.mdpi.com/article/10.3390/cancers14163887/s1, Figure S1. Full length blots of Figure 3A. Figure S2. Full length blots of Figure 3B. Figure S3. Change in the GRP78 concentration of exosomes contained in sera collected from gastric cancer patients according to the cancer stage. Table S1. Densitometry readings/intensity ratio of each band for blots of Figure 3A. Table S2. Densitometry readings/intensity ratio of each band for blots of Figure 3B. Table S3. Patient profile of measured samples. Supplementary data.
