# Supplementary material for: Ultrasensitive Detection of GRP78 in Exosomes and Observation of Migration and Proliferation of Cancer Cells by Application of GRP78-Containing Exosomes
Source: Cancers (Basel). 2022 Aug 11;14(16):3887. doi: 10.3390/cancers14163887 (PMC9405752; doi:10.3390/cancers14163887)
Supplement: Supplementary file 1 [file cancers-14-03887-s001.zip › cancers-1818438-supplementary.pdf]

cancers

MDPI

Supplementary Materials

Ultrasensitive Detection of GRP78 in Exosomes and Observation of Cancer Stemness Promotion by Application of GRP78-Containing Exosomes

Naoko Tsurusawa, Kanako Iha, Akane Sato, Hsin-Yi Tsai, Hikaru Sonoda, Satoshi Watabe, Teruki Yoshimura, Deng-Chyang Wu, Ming-Wei Lin and Etsuro Ito

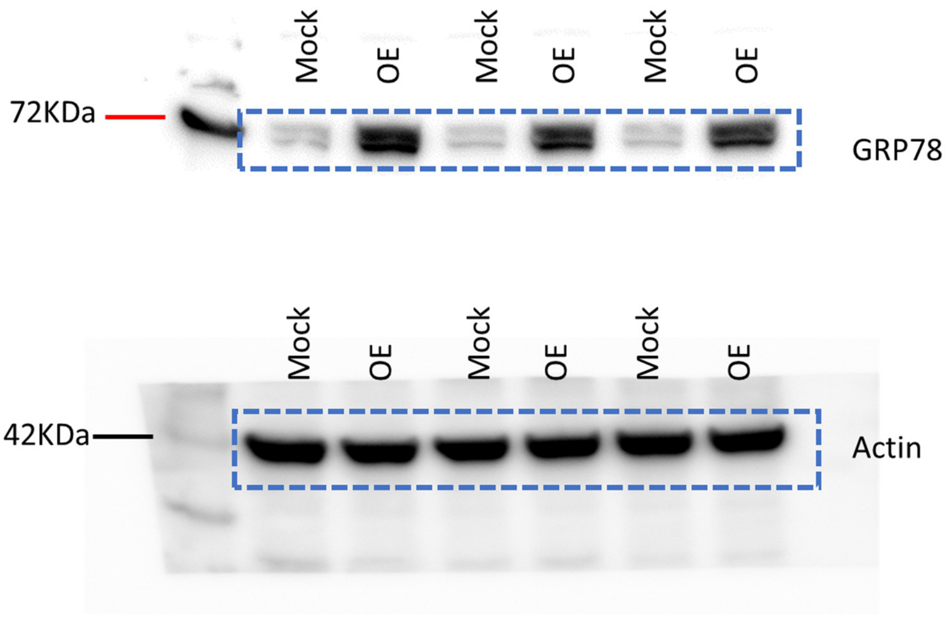

Figure S1. Full length blots of Figure 3A.

Table S1. Densitometry readings/intensity ratio of each band for blots of Figure 3A.

Cell Line	GRP78	Actin	GRP78/Actin
AGS GRP78-Mock	4607.296	12616.761	0.36517265
AGS GRP78-OE	23168.54	11594.368	1.99825803
AGS GRP78-Mock	3880.598	12325.69	0.3148382
AGS GRP78-OE	21103.49	13228.468	1.59530862
AGS GRP78-Mock	4697.104	13578.104	0.34593225
AGS GRP78-OE	21788.66	12839.882	1.69695173

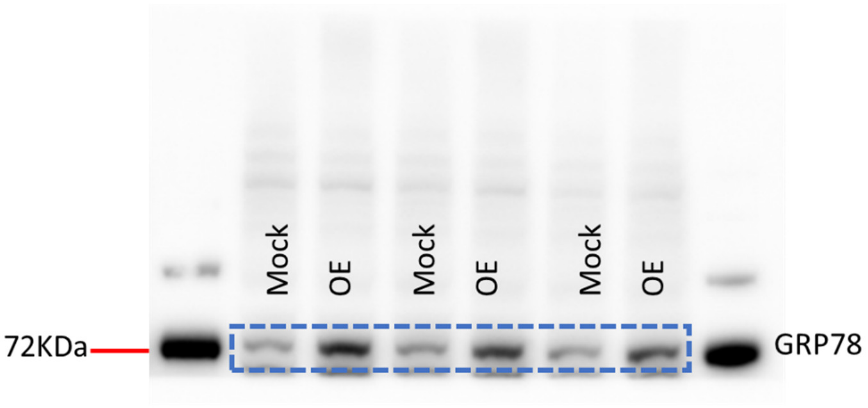

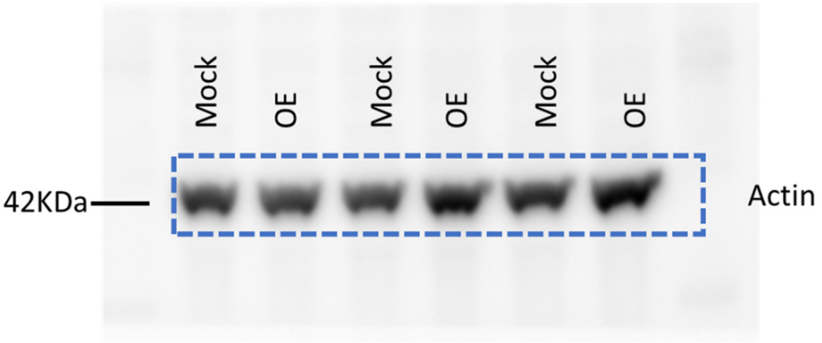

Figure S2. Full length blots of Figure 3B.

Table S2. Densitometry readings/intensity ratio of each band for blots of Figure 3B.

Cell Line	GRP78	Actin	GRP78/Actin
MKN45 GRP78-Mock	8479.518	11930.25	0.710758018
MKN45 GRP78-OE	21843.78	14097.4	1.549490448
MKN45 GRP78-Mock	9642.69	13206.59	0.730142355
MKN45 GRP78-OE	17954.59	15555.71	1.154212045
MKN45 GRP78-Mock	8045.832	14161	0.568168189
MKN45 GRP78-OE	15558.73	18187.76	0.855450597

Measurements of Gastric Cancer Patient Sera

We showed the data of ultrasensitive determination of GRP78 in exosomes obtained from gastric patient sera. The study was carried out in accordance with the recommendations of the principles and guidelines of the Declaration of Helsinki. All participants provided written informed consent to participate in the study. The protocols were approved by the academic research ethics review committees in Kaohsiung Medical University, Taiwan and in Waseda University, Japan [KMUH-IRB-20120176 (Kaohsiung Medical University to D.-C.W.), KMUHIRB-G(II)-20170028 (Kaohsiung Medical University to M.-W.L.), and 2019-076 (Waseda University to E.I.)]. A total of 34 serum samples was collected from patients with stage 1-4 gastric cancer at Kaohsiung Medical University (Table S3). Stage 1, n = 12; Stage 2, n = 9; Stage 3, n = 7 for; Stage 4, n = 6. The gastric cancer stage was determined by TNM staging. As control sera, we used 8 samples of reference sera (Cat # 06902, Nissui) as well as sera collected from 2 healthy patients at Kaohsiung Medical University (total number of control sera samples = 10).

Exosomes from the control sera and patient sera were extracted using Total Exosome Isolation Reagent (from serum) (Cat # 4478360, Thermo Fisher Scientific, Invitrogen). The sera were centrifuged at 2000× *g* for 20 min at room temperature to remove cells and cell debris. The supernatant was centrifuged at 10,000× *g* for 20 min at room temperature to

remove micro debris. A half volume of PBS was added to the supernatant, and 1/5 of the sample volume of the Total Exosome Isolation Reagent was added. The samples were incubated for 10 min at room temperature, and centrifuged at 10,000× *g* for 5 min at room temperature. The exosome-containing pellet was resuspended in PBS.

GRP78 in exosomes isolated from the sera of gastric cancer patients (TNM stages 1–4) and from control sera was measured by the ultrasensitive thio-NAD cycling ELISA (Figure S3). Data were scattered, and thus there were no significant differences. The GRP78 concentration in the exosomes tended to be higher in gastric cancer patients than in controls.

Table S3. Patient profile of measured samples.

Stage	T Stage	N Stage	M Stage	No.	Sex	Age	Location	Patho	Tumor Size (cm × cm)	Concentration of GRP78 in Exosome (pg/mL)
1	1b	0	0	G797	F	53	body	signet ring cell carcinoma grade3	2.4 × 1	8.50
1	1b	0	0	G822	F	51	subtotal gastrectomy	adenocarcinoma gradeIII	4.0 × 4.0	16.82
1	1a	0	0	G833	M	66	body	adenocarcinoma gradeIII	2.2 × 1.5	38.02
1	1	0	0	G860	M	75	antrum	large cell neuroendocrine carcinoma	0.5	12.72
1	3	1	0	G861	M	62	body	adenocarcinoma gradeII	2.8 × 1.8	6.60
1A	0	0	0	G791	M	54	antrum	adenocarcinoma gradeII	1.5 × 1.1	2.88
1A	1	0	0	G828	M	76	body	adenocarcinoma in situ	3.0 × 2.0	5.97
1A	2	0	0	G851	F	49	antrum	adenocarcinoma gradeIII	3 × 2.5	3.76
1A	1b	0	0	G857	M	60	body	adenocarcinoma gradeII	2.5 × 1.6	7.61
1A	2	0	0	G863	F	65	body antrum	adenocarcinoma gradeII	5.0 × 3.0	4.14
1A	1	0	0	G865	M	73	body	adenocarcinoma gradeII	1.5 × 1.4	10.26
1A-1B	2	0	0	G841	F	61	antrum	adenocarcinoma gradeII	2.2 × 2.0	5.34
2A	2	1	0	G799	M	88	antrum	adenocarcinoma gradeII	3.8 × 2.2	9.95
2A	2	1	0	G818	F	59	body	signet ring cell carcinoma grade3	1.2 × 1.0	20.36
2A	2	1	0	G826	F	66	body	adenocarcinoma gradeIII	2.0 × 1.7	8.62
2A	2	1	0	G845	F	58	greater	diffuse large B cell Lymphoma	2.0 × 1.8	18.21
2A	2	1	0	G859	M	66	antrum	adenocarcinoma gradeII	6 × 4	28.24
2B	4a	0	0	G813	M	67	subtotal gastrectomy	poorly-cohesive carcinoma grade3	4.0 × 3.5	5.97
2B	2	1	0	G832	M	63	body+antrum	adenocarcinoma gradeII	6.0 × 5.8	8.69
2B	3	0	0	G838	F	69	angle	adenocarcinoma gradeIII	3.5 × 2.5	2.57
2B	3	0	0	G842	M	53	antrum	adenocarcinoma gradeIII	2.5 × 1.7	3.13
3	4a	1	0	G817	F	82	antrum	adenocarcinoma gradeII	5.0 × 4.0	17.90
3	3	2	0	G840	F	76	antrum	adenocarcinoma gradeIII	0.3 × 0.1 × 0.1	7.74

3	3	2	0	G850	M	40	antrum	adenocarcinoma gradeII	9.5 × 4.0	3.76
3B	4a	2	0	G835	M	54	antrum	adenocarcinoma gradeIII	2.0 × 1.8	9.13
3B	no data	no data	0	G836	F	73	angle	adenocarcinoma gradeIII	3.3 × 2.1 × 0.9	4.77
3B	4a	0	0	G846	M	66	antrum	adenocarcinoma gradeII	5.8 × 4.5	26.16
3B	3	1	0	G848	M	84	antrum	poorly-cohesive carcinoma grade3	2.0 × 1.0	13.04
4	4a	2	0	G609	M	88	body	adenocarcinoma	6.0 × 5.1 × 1.2	18.84
4	3	2	0	G626			antrum	adenocarcinoma gradeI	0.4 × 0.2 × 0.2	5.09
4	4a	2	0	G825	M	51	antrum	adenocarcinoma gradeIII	7.0 × 5.0	55.44
4A	4b	0	0	G849	M	74	cardiac	Gastric carcinoma	1.0 × 0.5	4.65
4B	3	2	1	G844	M	84	body	adenocarcinoma gradeIII	3.5 × 1.7 × 0.4	3.76
4B	1	no data	1	G852	F	48	body	adenocarcinoma gradeIII	0.2 × 0.2 × 0.1	3.20

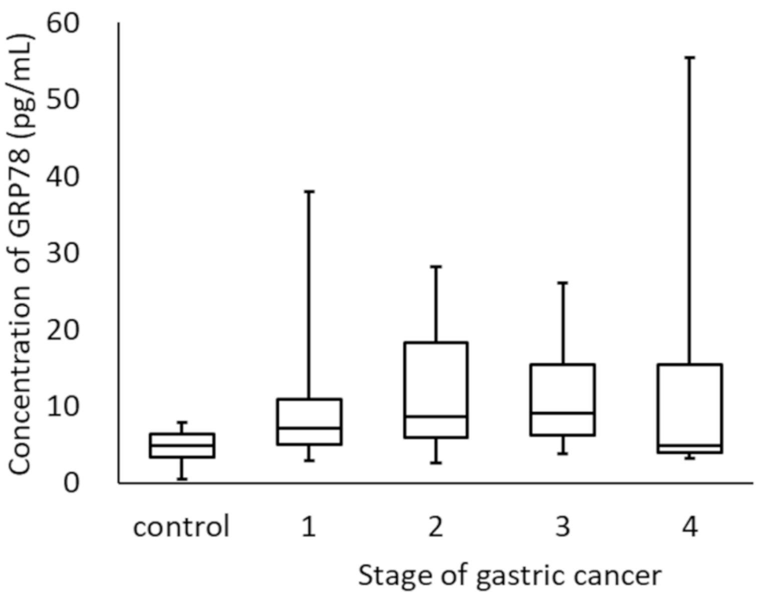

Figure S3. Change in the GRP78 concentration of exosomes contained in sera collected from gastric cancer patients according to the cancer stage. Control indicates data obtained from healthy subjects and control sera. Control, n = 10; Stage 1, n = 12; Stage 2, n = 9; Stage 3, n = 7; and Stage 4, n = 6. Data are expressed as mean ± SD. Cancer stage determined by TNM staging.
